# Visualization-driven digital technologies in oncology: current applications, technological advances, and future directions

**DOI:** 10.3389/fonc.2026.1824142

**Published:** 2026-08-20

**Authors:** Xiaoxia Duan, Yuxin Guo, Yanyan Yu, Sheng Li, Yujie Sun

**Affiliations:** 1 College of Future Technology, Peking University, Beijing, China; 2 Beijing Zestbridge Media Technology Co., Ltd., Beijing, China

**Keywords:** artificial intelligence, augmented reality, digital twin, oncology, visualization-driven digital technologies

## Abstract

Precise anatomical understanding and spatial cognition are fundamental to effective oncologic management. While traditional two-dimensional imaging serves as the diagnostic standard, it often lacks the intuitive depth required for complex decision-making. This review synthesizes the role of visualization-driven digital technologies in oncology—including AI-driven 3D reconstruction, augmented reality (AR), multimodal fusion, radiomics, and digital twins—as cognitive interfaces across the entire continuum of cancer care. We comprehensively review current applications, ranging from deep learning-enhanced early screening and segmentation in pre-treatment planning to AR-guided surgical navigation and adaptive radiotherapy during treatment. Furthermore, we highlight the emerging utility of radiomics in post-treatment surveillance and the humanistic value of immersive visualization in patient education for anxiety reduction. Despite these advancements, clinical integration remains impeded by challenges related to intraoperative precision stability, economic feasibility in resource-limited settings, and data privacy risks associated with biometric re-identification. Looking ahead, we propose that the future of oncologic visualization lies in the convergence of physics-informed deep learning for dynamic motion correction, cloud-based edge computing for cost democratization, and privacy-preserving collaborative learning strategies. Current advances in visualization technologies support the transition from passive anatomical viewing toward more interactive, data-driven clinical decision support, though the integration of these approaches into comprehensive predictive models requires further prospective validation.

## 1 Introduction

Medical visualization refers to the computational techniques that transform multidimensional clinical data into interpretable spatial and anatomical representations, serving as the foundational interface for clinical decision-making (1, 2). This technology provides the essential cognitive bridge upon which downstream applications—including image analysis, computer-aided diagnosis, radiomics, and predictive modeling—extract quantitative features and generate clinical insights, while artificial intelligence serves as the enabling methodology and digital twins represent comprehensive predictive simulation frameworks (3, 4). Visual analytics in medical informatics is established as the integration of analytical models with interactive visual interfaces to support clinical interpretation (5). Under this paradigm, radiomics and digital twins, while fundamentally analytical frameworks, are included when they project quantitative results onto spatial anatomical representations. Accordingly, this review adopts the broader lens of visualization-driven digital technologies in oncology—encompassing both core visualization systems and computational or analytical technologies whose clinical outputs are communicated through visual interfaces. The complexity of cancer diagnosis and treatment poses unprecedented challenges to modern oncology. Throughout tumor progression and treatment, each patient generates vast, complex datasets, including computed tomography (CT) scans, magnetic resonance imaging (MRI) findings, pathological sections, and genomic profiles (6). Interdisciplinary cancer diagnosis and treatment plans require collaborative formulation of solutions by professionals from multiple disciplines (7). Therefore, there is an urgent need for flexible and interactive visualization of patient data to provide physicians and other healthcare professionals with maximum information.

Numerous previous studies have demonstrated significant interobserver variability among clinicians in image interpretation. Specifically, interobserver variability in one-dimensional target lesion measurements according to the Response Evaluation Criteria in Solid Tumours (RECIST) can lead to discrepant overall response classifications in up to 12% of cases, with variability in the sum of longest diameters reaching 24% (8). In contrast, when moving to three-dimensional assessment, substantial interobserver variability persists in postsurgical tumor bed delineation on CT scans, with segmentation consistency highly dependent on cavity visualization scores and tumor bed volume (9). Despite continuous advancements in diagnostic imaging technologies, the inherent subjectivity of interpretation remains a considerable barrier to individualized treatment strategies. Thus, translating complex oncologicl data into actionable visualizations is an imperative and unmet clinical need.

Artificial intelligence (AI)-driven visualization platforms enable clinicians to assess tumor location and characteristics, thereby enhancing surgical feasibility. For example, platforms integrating dynamic contrast-enhanced MRI (DCE-MRI) can automatically generate three-dimensional renderings of tumors and surrounding structures through AI. This approach clearly quantifies tumor morphology and inter-tissue distances, improving surgical precision (10). In medicine, digital twins represent individuals by integrating multidimensional and multimodal data, enabling predictive simulation and clinical decision-making (11). In oncololgy, digital twin technology has demonstrated the capability to integrate extensive datasets to simulate radiographic images and oncological responses for predicting post-chemotherapy outcomes (12). Similar predictive modeling has been applied across diverse fields, including migraine and dementia management (11, 13, 14). These technologies are employed throughout the cancer care continuum. Applications range from 3D visualization navigation systems that reduce surgical complications to virtual reality platforms allowing patients to explore their own anatomical structures. By transforming complex medical data into intuitive visual representations, these tools enhance comprehension and treatment adherence. AI-driven visualization holds promise for applications in preoperative prediction, precision therapy, post-treatment care, and prognostic assessment. While medical visualization encompasses a broad spectrum of computational tools, this review focuses specifically on technologies that transform multidimensional oncologic data into spatial, anatomical, or interactive visual representations. These approaches serve as critical interfaces between complex datasets and human cognition, distinguishing them from purely algorithmic prediction models without visual output.

This narrative review was conducted through a comprehensive literature search of PubMed, Web of Science, and Scopus databases, focusing primarily on publications from 2015 to 2025 to capture recent technological advances while including foundational studies where relevant. Search strategies combined keywords related to visualization technologies (“medical visualization”, “3D reconstruction”, “augmented reality”, “hyperspectral imaging”, “digital twin”, “radiomics”) with oncologic terms (“cancer”, “oncology”, “surgical navigation”, “radiotherapy”, “endoscopy”). Studies were included if they reported quantitative clinical outcomes of visualization technologies in cancer screening, treatment planning, intraoperative guidance, post-treatment surveillance, or patient education. Priority was given to randomized controlled trials, large multicenter cohorts, and systematic reviews, while conference abstracts and non-peer-reviewed preprints were excluded. Technologies were considered within the scope of this review when their primary clinical function involves generating spatial, anatomical, or immersive visual representations that directly facilitate clinical interpretation and decision-making, rather than producing purely numerical or statistical outputs. The inclusion of four major technology categories is justified by their distinct visual contributions to oncological care: AI-enhanced 3D reconstruction and multimodal fusion generate spatial anatomical models essential for pre-treatment planning; augmented reality systems provide real-time visual overlays for intraoperative spatial guidance; visual implementations of analytical frameworks like radiomics and digital twins translate quantitative data into interpretable anatomical representations for post-treatment surveillance; and virtual reality platforms create immersive visual environments that facilitate patient education and communication. This taxonomy is organized by the sequential phases of cancer management, with each technology category corresponding to specific clinical functions in diagnosis, treatment, surveillance, and patient care.

**Figure 1:**
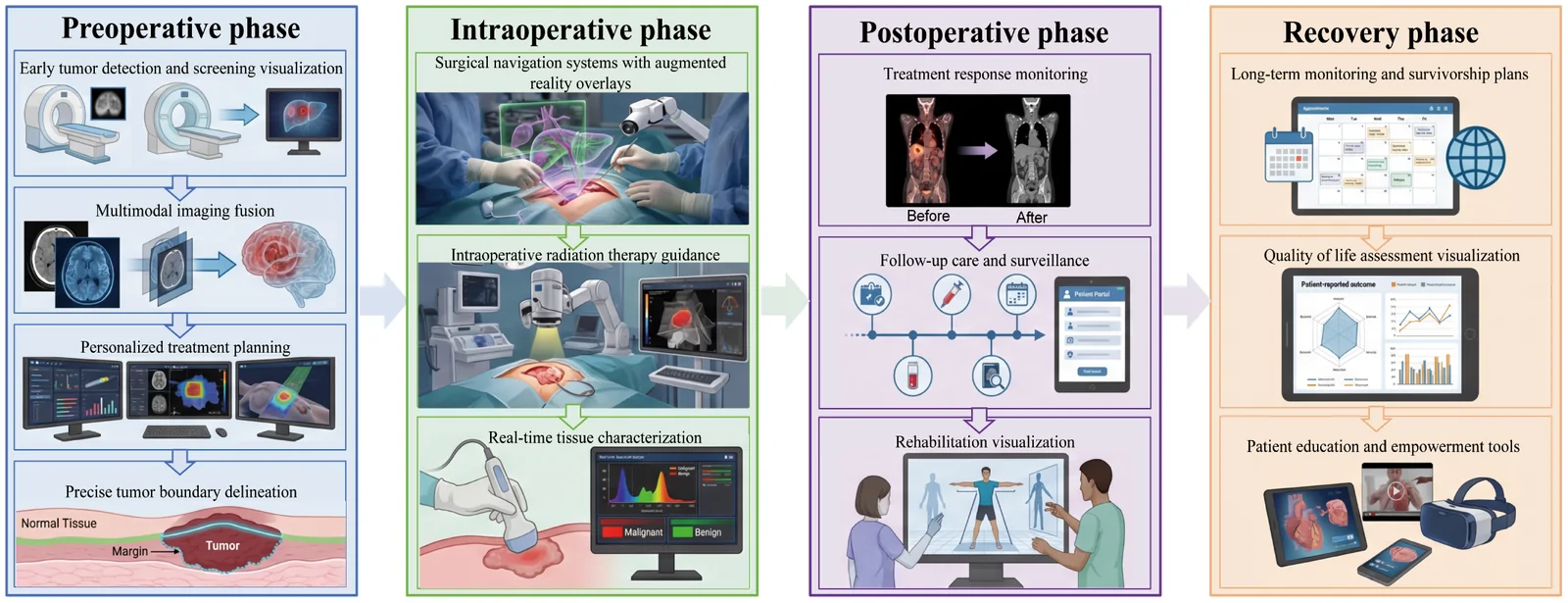
Applications of visualization-driven digital technologies in oncology throughout the cancer care continuum. This framework illustrates how visualization-driven digital technologies support clinical decision-making across four sequential phases. The Preoperative Phase encompasses AI-enhanced tumor detection, multimodal imaging fusion, and 3D treatment planning for precise boundary delineation. The Intraoperative Phase covers AR-guided surgical navigation, image-guided radiotherapy, and real-time tissue characterization. The Postoperative Phase includes radiomics-based treatment monitoring, surveillance workflows, and rehabilitation visualization. The Recovery and Survivorship Phase incorporates long-term monitoring tools, quality-of-life assessment, and immersive patient education platforms.

This review examines visualization-driven digital technologies across the cancer care continuum through a systematic framework that categorizes applications by technological sophistication—from AI-enhanced image reconstruction and multimodal fusion to real-time AR navigation and predictive digital twins—and by therapeutic objective, encompassing diagnostic screening, treatment planning, intraoperative guidance, post-treatment surveillance, and patient education (**Figure 1**). By synthesizing evidence from randomized controlled trials, large-scale multicenter cohorts, and systematic reviews (**Table 1**), we evaluate clinical efficacy, identify implementation barriers, and propose future research directions for researchers, clinicians, and healthcare administrators.

**Table 1:** Summarizes representative studies across this classification framework.

Therapeutic phase	Technology category	Sophistication level	Representative study	Sample size	Key clinical outcome
Pre-treatment Screening	DL-enhanced CT reconstruction	AI-Enhanced Imaging	Cao et al. (2023) - Multicenter validation	3,208 patients	Pancreatic cancer detection: 84.6% sensitivity, 99% specificity (17).
Treatment Planning	PET/MR fusion + ARM	Multimodal Integration	Flygare et al. (2023); Badve et al. (2025)	60 patients; Multi-center	Staging agreement κ: 0.35→0.57; PD-L1 detection ↑31% (18) (20).
Intraoperative Navigation	AR surgical guidance	Real-time Interactive	Ma et al. (2025) - Multicenter RCT	120 patients	Spinal surgery accuracy: 98% vs 91.7% (p<0.05) (28).
Post-treatment Surveillance	3D radiomics + AI	Predictive Modeling	Yu et al. (2025) - Multicenter cohort	1,199 patients	Breast cancer recurrence prediction: AUC 0.90 (38).
Patient Communication	VR/3D animation	Immersive Tools	Ye et al. (2023) RCT; Alimonaki et al. (2025) systematic review	Multi-center; 29 studies	Training completion: 63%→91%; 86% studies showed anxiety reduction (44).

## 2 AI-driven visualization in pre-treatment oncology

Early diagnosis is crucial for the prognosis of cancer patients. Timely and accurate diagnosis often leads to better therapeutic outcomes, particularly for patients with early-stage tumors, where curative treatment may be achievable (15). However, conventional two-dimensional imaging alone often poses challenges in screening early-stage disease. Modern medical visualization technologies can transform raw data or pathological pixel information into actionable spatial cognition, thereby reducing inter-observer variability and shortening the pathway from suspicion to definitive treatment (**Figure 1**, Preoperative Phase).

In lung cancer screening, a study using low-dose computed tomography (LDCT) demonstrated that three-dimensional deep learning-based image reconstruction reduced image noise inherent in conventional two-dimensional imaging. This approach improved both nodule detection rates and measurement accuracy compared to two-dimensional imaging alone (16). Similarly, in pancreatic cancer, a recent deep learning system trained on non-contrast CT scans identified early parenchymal density changes imperceptible to the human eye, achieving a sensitivity of 84.6% and specificity of 99% in a large-scale real-world validation cohort (17). Notably, the deep learning system additionally identified 26 patients with pancreatic lesions that were not detected during initial standard diagnostic workup (17). These cases demonstrate that pre-symptomatic visualization technologies, employed during early cancer screening or prior to biopsy, have the potential to alter subsequent clinical management pathways.

Once a lesion is identified, multimodal imaging fusion is often relied upon to characterize morphological, functional, and molecular phenotypes in order to determine tumor staging and refine the diagnostic workflow for subsequent treatment planning. A comparative study of regional imaging datasets in oropharyngeal squamous cell carcinoma revealed that PET/MR visual reconstruction improved interobserver agreement for tumor staging from κ=0.35 to 0.57. The addition of PET increased the Dice similarity coefficient (DSC) from 0.68 to 0.83 (*p* < 0.001) (18, 19). In the pathology domain, augmented reality microscopy (ARM) directly overlays AI-computed PD-L1 combined positive score (CPS) onto slide images, improving pathologist diagnostic concordance by 26% and increasing the detection rate of immunotherapy-eligible cases by 31% (20). In gastrointestinal oncology, hyperspectral imaging and spectrum-aided visualization technologies have emerged as promising approaches for enhancing endoscopic diagnostics. The Spectrum-Aided Vision Enhancer (SAVE) algorithm enables computational conversion of standard white-light endoscopic images into spectrally enriched representations without requiring specialized hardware, demonstrating superior mucosal visualization in capsule endoscopy (21). When integrated with deep learning architectures, SAVE-enhanced images consistently outperform conventional white-light imaging in early cancer detection and gastrointestinal disorder classification, achieving F1-scores up to 96% for lesion identification (22). Thus, synthetic visualization of multi-omics data enhances diagnostic consistency and biomarker quantification capabilities, both of which are prerequisites for precision treatment selection.

Targeted treatment planning benefits most significantly from 3D/4D rendering. In radiation oncology, deep learning-based automatic segmentation of organs at risk (OARs) on planning CT has shown promise for reducing contouring time while maintaining geometric accuracy. When the same framework was applied to tumor beds following breast-conserving surgery, inter-observer volume variability (Δinter) decreased from 37.0% to 15.6% (*P* = 0.009) for tumor beds with volumes ≥25.2 cm³ (9). For surgical candidates, patient-specific 3D-printed liver models reduced surface error to 1.3% (≈0.8 mm) and increased the R0 resection rate from 72% to 91% (23). These preoperative visualization technologies translate anatomical details into more precise therapeutic interventions. Supporting these individual studies, recent meta-analytic evidence provides stronger clinical validation. A systematic review and meta-analysis of randomized controlled trials demonstrated that AI-assisted visualization systems significantly improve adenoma detection rates by 24% compared to conventional colonoscopy, offering Level I evidence for integrating these technologies into routine cancer screening programs (24).

Throughout the screening-to-planning continuum, large-scale validation studies demonstrate three tangible advantages: earlier lesion detection, reduced inter-observer variability, and optimized treatment pathways. Notably, AI-enhanced imaging reconstruction and multimodal fusion technologies show the strongest evidence base, with multicenter trials consistently reporting improved diagnostic accuracy and staging agreement. However, the transition from single-center feasibility studies to large-scale RCTs remains incomplete for fully automated planning systems, indicating areas requiring further validation. Besides technical performance metrics, these visualization technologies show unique value in clinical interpretation by reducing the cognitive load of reconstructing 3D anatomical structures in the mind from multiple 2D image sequences. This helps multidisciplinary teams reach more consistent diagnostic consensus and treatment plans.

## 3 Intra-treatment visualization applications

As clinical priorities shift from diagnostic planning to active intervention, visualization technologies transition from static anatomical mapping to dynamic, real-time spatial guidance during surgical resection and precision radiochemotherapy for patients with advanced cancer. Current research focuses predominantly on several domains, including augmented reality surgical navigation systems, positron emission tomography-guided adaptive radiotherapy, and interventional ultrasound fusion techniques (**Figure 1**, Intraoperative Phase) (25–27).

Head-mounted AR displays overlay preoperative 3D models onto the surgical field, enabling clinicians to simultaneously view precise datasets and real-world anatomy. This reduces the need to repeatedly reference external monitors during surgery. A clinical randomized controlled trial demonstrated that AR surgical navigation systems achieved significantly superior accuracy for pedicle screw placement in spinal fixation surgery compared to conventional CT-guided procedures (98% vs. 91.7%, P<0.05) (28). A prospective feasibility study (n=10) reported submillimeter-level AR registration accuracy in 3D laparoscopic and robotic liver resections (Dice coefficient 0.912 ± 0.052, corresponding to <1 mm target error) (29). A pooled analysis of 19 skull base studies (n=508) revealed a median target registration error of 0.82 ± 0.37 mm for AR navigation (30).

Diagnosing locoregional metastasis during treatment remains challenging. This underscores the importance of assessing treatment efficacy and adjusting therapeutic regimens in a timely manner. AI-driven multimodal imaging holds promise for improving diagnostic accuracy. Integration of PET/MR dual-modality probes into the linear accelerator gantry serves as a “sentinel” at critical sites, enabling online monitoring of metabolic hotspots—those either not visualized on planning PET/CT or emerging during fractionated treatment. In a retrospective study of adaptive radiotherapy for head and neck cancer patients, adaptation significantly reduced the mean radiation dose to critical structures. While maintaining target volume coverage, MR-guided adaptive replanning decreased the cumulative mean dose to the parotid glands by 4.1% and to the submandibular glands by 0.1% (31). Offline PET-guided biology-guided radiotherapy (BgRT) also achieved tighter isodose distributions and reduced organ-at-risk doses while maintaining treatment completion rates. This corroborates the therapeutic window benefits conferred by metabolic imaging (32).

Furthermore, multimodal microscopy imaging probes integrated with deep learning methods enable automated cancer detection and spectral-guided femtosecond ablation. Multimodal nonlinear images of thin tissue sections are captured via endomicroscopy, followed by visual assessment by experienced pathologists to categorize epithelium, tumor stroma, necrosis, tumor, other tissues, and background, facilitating the differentiation of malignant tissues (33). A study of 124 patients undergoing radiofrequency and microwave ablation found that AI-based deformable image registration (DIR) combined with auto-segmentation for ablative margin quantification revealed that 45% of patients achieved ablation margins ≥5 mm (34). An ablation margin of 0 mm was identified as a significant independent predictor of local disease progression, with DIR combined with automatic segmentation demonstrating a subdistribution hazard ratio of 23.3 (95% CI: 10.8–50.5, P<0.001). Apart from small case series, aggregated data confirm the extensive clinical value of intraoperative visualization. A systematic review of three-dimensional computed tomography bronchovascular imaging in thoracic oncology demonstrated that, compared with conventional two-dimensional preoperative planning, preoperative three-dimensional visualization significantly reduces procedural complexity and enhances surgical precision (35). Overall, AR-guided navigation demonstrates the most robust clinical evidence with consistent sub-millimeter accuracy, while adaptive radiotherapy shows promising dose reduction and AI-driven ablation remains in feasibility phases. Standardized reporting frameworks are needed to address outcome heterogeneity across studies. The clinical impact of intraoperative visualization technology not only improves accuracy but also streamlines workflow and enhances surgical safety. By turning complex spatial judgments into shared visual references, these visualization tools reduce the cognitive load between different doctors during key surgical steps, helping the surgical team make timely decisions and ultimately lowering the risk of complications.

## 4 Post-treatment visualization application

With the advancement of artificial intelligence and imaging technologies, the role of imaging in the survivorship phase has shifted from treatment guidance to early recurrence surveillance and late toxicity monitoring. When integrated with visual analytics, radiomics-based prognostic tools translate quantitative imaging features into spatially interpretable risk assessments, showing broad potential in oncologic prognosis prediction (**Figure 1**, Postoperative Phase). Radiology-based visualization models can effectively predict hepatic and lymph node metastases. For lymph node prediction, the AUC reached 0.85 (36). Fan et al. developed a radiomics-based integrated model to predict the risk of postoperative recurrence in stage II colon cancer patients (37). When radiomics was combined with clinicopathological prognostic factors for recurrence prediction, the AUC reached 0.954 in the training cohort and 0.906 in the validation cohort, demonstrating excellent predictive performance (37). Yu et al. conducted a retrospective multicenter study of 1,199 patients, introducing a multimodal MRI and AI-driven 3D deep learning model to predict recurrence risk in non-metastatic breast cancer patients. The 3D-MMR model achieved an AUC of 0.90 for two-year disease-free survival prediction, reflecting the model’s robustness (38). In HPV-negative head and neck cancer patients, data from 12 pooled cohorts showed that post-treatment 18F-FDG PET/MR performed 12 weeks after chemoradiotherapy resulted in clinical management changes in 27% of patients. The majority of these changes were triggered by detection of persistent lymph nodes or second primary tumors not identified on contrast-enhanced CT. This management change rate exceeded the 10% threshold established by ESMO for cost-effective imaging surveillance, further cementing the status of PET/MR visualization as the default monitoring modality for high-risk metastatic sites (39). In longitudinal studies utilizing radiological features, 13 delta radiomics features (DRFs) demonstrated significant changes in treatment response, enabling patients to be stratified as good or poor responders. The predictive capability for treatment response achieved an AUC of 0.94, suggesting that DRFs may play a pivotal role in optimizing therapeutic strategies (40). Radiomics-based models consistently achieve robust predictive utility (AUC >0.85) in large retrospective cohorts, though prospective validation and head-to-head comparisons with established prognostic tools remain limited. Besides prediction accuracy, visualization-based prognostic tools help with clinical decision-making by turning complex radiomics features into easy-to-understand risk assessments. This allows doctors to come up with more targeted monitoring strategies.

## 5 Patient communication and education visualization

### 5.1 3D-animation-based patient education

Enhancing patient understanding of their disease and treatment plans can improve clinical decision-making, patient engagement, adherence, and health outcomes, while also alleviating patient anxiety. Previous research has found that 80% of information conveyed by healthcare professionals during outpatient consultations may be forgotten (41). This may be attributed to multiple factors: variations in patients’ educational levels and knowledge backgrounds; brief but highly technical terminology used by physicians; and evolving treatment modalities—all of which render physician-patient communication and patient education increasingly challenging. A study promoting adherence to active breathing exercises for postoperative cancer rehabilitation found that, compared to traditional face-to-face physician education, the animated education group exhibited fewer postoperative pulmonary complications and demonstrated superior effectiveness (42). Additionally, animated visualization can improve early mobilization in preoperative rectal and gynecological oncology patients (43). Through effective preoperative mobilization interventions, patients demonstrated significantly increased daily step counts postoperatively, and the brief preoperative intervention enhanced adherence to postoperative mobilization. In a multicenter randomized controlled trial of patients with metastatic colorectal cancer, a 3-minute three-dimensional animation explaining pelvic floor muscle mechanisms increased training completion rates from 63% to 91% (*p* = 0.002) compared to verbal instruction alone, and reduced LARS scores from 30.87 ± 3.77 to 25.48 ± 2.16 (*p* < 0.001) over 3 months (44). These quantitative data demonstrate that molecular-level visualization can translate abstract physiology into actionable skills while reducing instructional time without requiring additional personnel. Realistic 3D animations enable patients to fully comprehend disease-related knowledge and reinforce understanding during postoperative follow-up visits. They effectively facilitate the formation of durable memories in patients and enhance treatment adherence continuity (**Figure 1**, Recovery & Survivorship Phase).

### 5.2 Immersive pre-operative communication and anxiety control

Perioperative care often involves addressing patient stress and anxiety, with preoperative anxiety being associated with postoperative pain, delayed wound healing, and increased risk of complications. Therefore, effective identification and management of preoperative anxiety can contribute to improved postoperative outcomes. Traditional interventions such as music therapy, acupuncture, and relaxation techniques have been helpful in alleviating preoperative anxiety. In recent years, virtual reality (VR), owing to its visualizable and immersive characteristics, has demonstrated effectiveness in reducing patient anxiety levels. A systematic review incorporating 29 studies indicated that 86% of the studies showed significant reductions in preoperative anxiety levels following VR interventions (45). A VR-based interventional trial in brain tumor patients revealed that remote VR interventions using devices self-administered by patients at home had positive effects on patient stress and anxiety (46). Furthermore, VR interventions can enhance pediatric oncology patients’ understanding of radiation therapy procedures and reduce anxiety in both children and their parents (47).

Tamsin et al. introduced a proof-of-concept extended reality (XR) environment that displays genomic information from multiple tumor sites through 3D tumor models, accurately conveying information such as disease etiology to patients via 3D imagery, thereby enhancing patient understanding of their disease and treatment while strengthening trust in the diagnosis through XR technology (48). Patient-centered tools, particularly VR-based anxiety interventions, demonstrate consistent clinical benefits across multiple trials, yet standardized outcome measures and longer follow-ups are required to establish durability. These research results show that visualization has a unique role in patient communication. It can turn abstract physiological concepts into concrete models, reduce understanding gaps between different patients, and help patients participate more effectively in shared decision-making, which in turn improves treatment adherence and clinical outcomes.

## 6 Challenges and limitations of medical visualization in oncology

Medical visualization technologies serve as cognitive interfaces that translate complex oncological data into spatial representations for clinical decision-making, yet their integration remains limited by a persistent gap between technical validation and demonstrated patient benefit. Much of the current literature relies on surrogate technical metrics—Dice coefficients, AUC values, and registration errors—which indicate algorithmic performance but do not necessarily translate to improved survival or reduced morbidity. Additional barriers include insufficient precision, economic constraints, and data privacy concerns.

First, the rapid movement of visualization cameras during surgical procedures can induce latency in AR systems, which may compromise the accuracy of alignment and increase the complexity of localization (29). Moreover, prolonged viewing of AR displays can lead to visual fatigue, thereby undermining surgical safety. In a feasibility study of image-guided surgery for pediatric osteosarcoma using a porcine cadaver model, eight registration procedures yielded a mean target registration error of 6.78 ± 1.33 mm, indicating that further improvements in precision are required before routine clinical implementation (49). These discrepancies indicate that further improvements in precision are imperative before the clinical translation and widespread implementation of such technologies. Clinical experience illustrates these precision gaps. In AR-guided spinal surgery, while overall pedicle screw accuracy improved to 98% versus 91.7% for conventional methods, surgeons still required fluoroscopic verification in complex segments due to registration drift (28). Similarly, skull base procedures report submillimeter target registration errors under controlled conditions, yet intraoperative tissue deformation and cerebrospinal fluid leakage frequently necessitate system recalibration (30). The clinical consequences of spatial inaccuracy are particularly evident in thermal ablation, where 0-mm margins were associated with a subdistribution hazard ratio of 23.3 for local disease progression (95% CI: 10.8-50.5), demonstrating that visualization precision directly impacts patient outcomes (34).

Second, economic feasibility warrants attention. The viability of high operational costs in practical implementation, particularly in resource-limited settings, remains uncertain. The implementation of high-fidelity visualization systems, especially those involving VR, AR, and 3D printing, requires substantial initial capital expenditure. This encompasses the procurement of high-performance computing workstations, proprietary software licenses, and specialized hardware peripherals such as head-mounted displays or haptic feedback devices (50). Beyond direct procurement costs, the “hidden costs” associated with workflow integration and maintenance are frequently underestimated. Effective utilization of these tools demands a steep learning curve, requiring sustained investment in specialized training for radiologists and surgeons, as well as the employment of technical support personnel to manage data segmentation and system calibration (51). These cost barriers create uneven adoption patterns across healthcare settings. While systematic reviews document the potential benefits of 3D printing in surgical planning, material and personnel costs remain prohibitive for routine use in many institutions (50). Time-motion studies show that 3D-printed models can reduce operative time by 23–62 minutes per case in orthopedic and maxillofacial procedures, yet these efficiency gains often fail to offset implementation costs without dedicated reimbursement pathways (51). Consequently, advanced visualization technologies remain concentrated in high-volume academic centers, while community hospitals limit their use to exceptional cases, creating disparities in access to precision-guided oncological care. Apart from cost considerations, multiple translational barriers frequently result in failed implementation. External validation remains problematic: many AI-driven visualization algorithms perform excellently on internal datasets, yet their performance declines substantially when deployed across different institutions, imaging device manufacturers, or patient populations. Workflow integration also presents substantial challenges, as implementing these systems often requires substantial revisions to established clinical pathways, additional quality control procedures, and prolonged operational time during the learning curve for clinical staff—burdens that busy oncology departments struggle to bear. Algorithm performance is highly sensitive to minor variations in imaging acquisition protocols or software versions, further aggravating reproducibility concerns. The regulatory pathway for continuously adaptive visualization systems remains unclear, and the absence of dedicated reimbursement codes for visualization-guided surgery creates a persistent structural barrier independent of clinical evidence.

In addition, the proliferation of medical visualization technologies inevitably exacerbates the vulnerability of digital patient privacy. Unlike traditional DICOM images where anonymization can be achieved by stripping metadata, high-fidelity 3D surface reconstructions—particularly in head and neck oncology—retain intrinsic biometric features (such as facial topology) that pose a significant risk of patient re-identification even after de-identification protocols (52). Moreover, the increasing reliance on cloud-based rendering and wireless transmission for mobile or VR/AR visualization expands the data attack surface, exposing sensitive oncological data to potential interception during real-time streaming. Therefore, establishing robust encryption standards for 3D geometric data and developing “privacy-preserving” rendering algorithms remains a critical, yet unresolved, ethical imperative (53). These privacy concerns have immediate operational consequences. Face-recognition software has successfully re-identified supposedly anonymized MRI participants from 3D facial reconstructions, challenging conventional de-identification protocols for volumetric imaging data (52). In practice, hospital IT departments often restrict wireless AR headsets and cloud-based rendering services due to network security requirements, creating a conflict between data protection and system functionality. Multi-center oncology trials requiring shared 3D models for collaborative treatment planning face complex data-use agreements that can prohibit the cross-institutional data sharing necessary for AI algorithm improvement (20).

Collectively, these impediments—spanning precision limitations, economic constraints, translational barriers, and data privacy risks— underscore a fundamental reality: technical sophistication alone is insufficient for clinical integration. To translate the promise of medical visualization into routine oncological practice without financially overburdening healthcare systems or eroding patient trust, synchronous advancements are imperative across the domains of physiological motion modeling, value-based procurement models, privacy-by-design engineering, and regulatory science.

## 7 Conclusions and future directions

Existing evidence reveals promising directions for overcoming current technical limitations. While augmented reality navigation systems achieve submillimeter accuracy under controlled conditions, real-time compensation for soft tissue deformation remains a major technical hurdle. High implementation costs also drive the need for alternative deployment frameworks. The digital twin concept has proven feasible for cancer treatment simulation, and radiomics models have shown robust predictive performance in large patient cohorts. Nevertheless, whether integrating these two modalities can optimize clinical outcomes remains to be validated in prospective studies. Privacy-preserving federated learning offers a viable solution for multicenter AI research collaboration, yet its practical implementation framework is still in its infancy. Despite their technological diversity across different cancer types and clinical contexts, these visualization-driven digital technologies share a unifying clinical objective: reducing subjective interpretation, standardizing complex workflows, and bridging the gap between abstract data and actionable human decisions.

In summary, current evidence demonstrates that visualization-driven digital technologies in oncology can improve specific measurable outcomes—including earlier lesion detection, reduced interobserver variability in treatment planning, submillimeter surgical guidance accuracy, and enhanced radiomics-based prognostication. However, whether these technical improvements consistently translate into improved patient survival and quality of life across diverse clinical settings requires further prospective validation in routine practice.
